# Adiposity, Body Composition, and Cardiometabolic Outcomes in Women with and Without Gestational Diabetes

**DOI:** 10.3390/metabo16100727

**Published:** 2026-09-29

**Authors:** Marietta Charakida, Cristina Gómez Fernández, Tanvi Mansukhani, Carolina Matta, Holy McMahon, Disha Bhojwani, Kypros H. Nicolaides

**Affiliations:** 1 Harris Birthright Research Institute, King’s College Hospital, London SE5 8BB, UK; cristinagofernandez@gmail.com (C.G.F.); tanvi.mansukhani@nhs.net (T.M.); carolina.jmatta@nhs.net (C.M.); holy.mcmahon@nhs.net (H.M.); disha.bhojwani@nhs.net (D.B.); kypros@fetalmedicine.com (K.H.N.); 2 Cardiovascular Imaging Department, School of Biomedical Engineering, King’s College London, London SE1 7EH, UK; 3 2nd Paediatric Department, P&A Kyriakou Hospital, National and Kapodistrian University of Athens, 115 27 Athens, Greece; 4 Faculty of Medicine, Universidad Complutense of Madrid, 28040 Madrid, Spain

**Keywords:** adiposity, gestational diabetes, body mass index, hypertension, dysglycaemia, steatosis

## Abstract

**Highlights:**

**What is the main question?**

- Does detailed body-composition assessment improve early postpartum cardiometabolic risk prediction beyond conventional anthropometric measures in women with and without prior gestational diabetes mellitus (GDM)?

**What are the main findings?**

- Women with prior GDM had greater overall and regional adiposity and a higher burden of dysglycaemia, hypertension, dyslipidaemia, and liver steatosis. Body-composition measures improved phenotyping but did not demonstrate superior discrimination compared with BMI and waist-to-height ratio.

**What is the clinical implication?**

- Simple anthropometric measures remain practical first-line tools for postpartum risk stratification, while body-composition assessment may provide complementary phenotypic information in selected women and research settings.

**Abstract:**

**Aims**: Gestational diabetes mellitus (GDM) is associated with increased long-term cardiometabolic risk. The value of body composition measures in predicting adverse cardiometabolic outcomes after GDM remains uncertain. **Methods**: In this prospective observational study, 757 women (300 controls and 457 with GDM in their index pregnancy) attended a postnatal assessment at a median of 5.8 (IQR 5.1–7.1) months after delivery. Anthropometric measures, biochemical markers, cardiovascular assessments, and body composition by bioelectrical impedance analysis were obtained. Outcomes included dysglycaemia, dyslipidaemia, hypertension, metabolic syndrome and liver steatosis. Multivariable logistic regression models evaluated associations and predictive performance. **Results**: Compared with controls, women with prior GDM had higher BMI, central adiposity, and fat mass (all *p* < 0.001). They also had a higher prevalence of dysglycaemia (42.9% vs. 16.7%), hypertension (30.2% vs. 18.7%), dyslipidaemia (6.3% vs. 3.0%), and liver steatosis (33.3% vs. 14%; all *p* < 0.05). BMI and waist-to-height ratio improved prediction of adverse outcomes beyond maternal characteristics alone. Body composition measures were associated with cardiometabolic outcomes but did not show superior discriminatory performance compared with conventional anthropometric measures. **Conclusions**: Women with prior GDM have a high burden of postpartum cardiometabolic abnormalities. While body composition provides additional biological insight, simple anthropometric measures remain the practical first-line tools for risk stratification.

## 1. Introduction

Gestational diabetes mellitus (GDM) is one of the most common metabolic complications of pregnancy, affecting up to 15–20% of pregnancies worldwide and increasing in prevalence globally [1,2]. Its rise parallels increasing maternal age, obesity, and shifting population demographics. Although GDM is often considered a transient condition, it is now well established that it identifies women at substantially increased risk of long-term cardiometabolic disease, including type 2 diabetes, hypertension, dyslipidaemia and cardiovascular disease [3,4,5,6].

The postpartum period represents a critical window for risk stratification and early intervention [7,8]. However, current clinical approaches rely largely on conventional anthropometric measures such as body mass index (BMI) and waist circumference, which may inadequately capture the complexity of adiposity and its relationship with metabolic risk [9]. In particular, BMI does not distinguish between fat and lean mass, nor does it reflect fat distribution, both of which are key determinants of insulin resistance and cardiometabolic health [10].

Emerging evidence suggests that body composition parameters—such as fat mass, fat-free mass, and regional adiposity—may provide a more precise assessment of metabolic risk [11,12]. Visceral and ectopic fat depots, including abdominal and intramuscular adipose tissue, have been shown to play a central role in the pathophysiology of insulin resistance and hepatic steatosis [13,14]. However, the extent to which these measures improve prediction beyond conventional anthropometric indices remains unclear, particularly in women with prior GDM, and few studies have directly compared these approaches across a broad range of cardiometabolic outcomes in the early postpartum period.

Therefore, the aim of this study was to compare anthropometric and body composition measures in women with and without GDM in their index pregnancy, evaluate differences in postpartum cardiometabolic outcomes, and determine whether body composition improves performance beyond conventional anthropometric indices.

## 2. Material and Methods

### 2.1. Study Population and Design

This was a prospective observational study conducted between October 2023 and November 2025 at the Fetal Medicine Unit, King’s College Hospital, London, UK. The study was undertaken under NHS Research Ethics Committee approval (REC reference 18/NI/0013; IRAS ID 237936), originally approved on 22 January 2018, under an existing research protocol and its subsequent amendments. Amendment 8, approved on 9 May 2025, specifically covered the body composition assessment performed at the postnatal visit. Antenatal recruitment was completed by 20 May 2025, ensuring that all participants had sufficient time to undergo the planned postnatal assessment within the study period, which ended on 1 November 2025. Only participants who had completed the scheduled postpartum assessment before database closure on 1 November 2025 were included in the current analysis. Consecutive women attending routine antenatal care, including ultrasound examinations at 12, 20, and 36 weeks’ gestation, were invited to participate. Women diagnosed with GDM in the index pregnancy were identified according to National Institute for Health and Care Excellence (NICE) criteria, based on a 75 g oral glucose tolerance test (OGTT). GDM was diagnosed if fasting plasma glucose was ≥5.6 mmol/L and/or the 2 h plasma glucose was ≥7.8 mmol/L [15].

Participants were invited to attend a standardized postnatal follow-up visit approximately 5–6 months after delivery. At this visit, maternal demographic characteristics, medical and obstetric history, and pregnancy outcomes were recorded, including age, ethnicity, parity, family history of diabetes, history of GDM, gestational hypertension, and preeclampsia. Socioeconomic status was recorded as the Index of Multiple Deprivation quintile, assigned according to the postcode of the participant’s home address [16]. Data on baseline maternal and pregnancy characteristics and outcomes were collected/verified from hospital maternity records or women’s general practitioners.

The study was conducted in accordance with the ethical principles of the Declaration of Helsinki and was approved by the National Health Service Research Ethics Committee (reference 18/NI/0013; Integrated Research Application System ID 237936). All participants provided written informed consent prior to participation.

### 2.2. Postnatal Biochemical Assessment

Anthropometric measurements, biochemical testing, and cardiovascular assessments were performed. All women underwent biochemical testing in maternal blood, including HbA1c (mmol/mol), serum triglycerides (mmol/L), serum high-density lipoprotein (HDL, mmol/L), serum creatinine (µmol/L), and urine albumin/creatinine ratio (uACR, mg/mmol). All laboratory assays were performed according to standard operating procedures. Serum low-density lipoprotein cholesterol (LDL, mmol/L) was calculated using the Friedewald equation: LDL = Total Cholesterol-HDL-(Triglycerides/2,2) [17]. Additionally, the GDM group attended fasting (≥8 h) and underwent a 75 g 2 h OGTT.

### 2.3. Adiposity and Body Composition

Anthropometric assessment at the postnatal visit included measurement of weight (kg), height (m), and waist circumference (cm) using standardized protocols. BMI (kg/m^2^) was calculated as weight divided by height squared and was classified as normal (18.5–24.9 kg/m^2^), overweight (25.0–29.9 kg/m^2^), obesity Class I (30.0–34.9 kg/m^2^), obesity Class II (35.0–39.9 kg/m^2^), or obesity Class III (≥40.0 kg/m^2^) [18]. Waist-to-height ratio (WHtR) was calculated as waist circumference divided by height, and a value > 0.5 was considered indicative of central adiposity.

Body composition was assessed using bioelectrical impedance analysis (BIA) with the BIA-ACC device (BioTekna S.r.l., Marcon, Venice, Italy). BIA is an effective, safe, non-invasive, and low-cost method for estimating body composition by passing a low-voltage alternating current through the body and measuring how different tissues conduct electricity based on their properties [19]. This method provides estimates of fat mass (FM, %), fat-free mass (FFM, %), total body water (TBW, %), intracellular water (ICW, %), extracellular water (ECW, %), and derived indices including the fat mass-to-fat-free mass ratio (FM/FFM). In addition, regional adiposity measures were obtained, including intramuscular adipose tissue (IMAT, kg), total adipose tissue (AT, kg), and abdominal adipose tissue (AAT, cm^2^). Measurements were performed during the postnatal visit in a standard clinical setting. Participants avoided physical activity for at least 2 h beforehand and were assessed in the supine position on a non-conductive surface, with legs slightly apart and arms not touching the body. Fasting, recent fluid intake, and bladder voiding were not systematically standardized before BIA assessment. Disposable electrodes (BioTekna) were placed on the right hand (metacarpal and wrist) and right foot (metatarsal and ankle).

### 2.4. Cardiometabolic Outcomes

Dysglycaemia was defined as the presence of prediabetes or type 2 diabetes mellitus. Prediabetes was defined according to American Diabetes Association criteria [20] as one or more of the following: HbA1c 39–47 mmol/mol, fasting plasma glucose (FPG) 5.6–6.9 mmol/L, or 2 h glucose during oral glucose tolerance test (OGTT) 7.8–11.0 mmol/L. Type 2 diabetes was defined using World Health Organization criteria (HbA1c ≥ 48 mmol/mol, fasting glucose ≥ 7.0 mmol/L, or 2 h glucose ≥ 11.1 mmol/L).

Dyslipidemia [21] was defined as the presence of the following: high triglyceride levels (defined as fasting serum triglycerides ≥ 1.7 or ≥2.3 mmol/L non-fasting), low HDL cholesterol < 1.2 mmol/L and/or use of lipid-lowering treatment.

Blood pressure was measured using standardized techniques [22]. Hypertension [23] was defined as systolic blood pressure ≥ 130 mmHg or diastolic blood pressure ≥ 80 mmHg, or current use of antihypertensive medication. Hypertension was further classified as Stage 1 (130–139/80–89 mmHg) or Stage 2 (≥140/90 mmHg).

Renal dysfunction was defined as the presence of microalbuminuria (uACR ratio ≥ 3 mg/mmol) and/or reduced eGFR (eGFR < 90 mL/min/1.73 m^2^), calculated by the 2009 Chronic Kidney Disease-Epidemiology Collaboration without the race coefficient [24].

Assessment of hepatic steatosis and liver stiffness was performed with Vibration Controlled Transient Elastography (VCTE) using a FibroScan^®^ Compact 530 (Echosens, Paris, France). Examination was considered suitable when at least 10 consecutive measurements were recorded, with an IQR/Median for stiffness measurement ≤ 30%. The XL probe was used for patients with a high BMI or when good quality measurements could not be obtained with the standard M probe. Both liver stiffness measurement (LSM, kPa) and controlled attenuation parameter (CAP, dB/m) were recorded. Liver steatosis was defined as CAP ≥ 248 dB/m and fibrosis as LSM ≥ 8 kPa. These thresholds have been validated predominantly in general adult populations and have not been specifically validated in early postpartum women [25]. The interval between the last meal and transient elastography examination was recorded. The potential influence of this interval on CAP and liver stiffness measurements had previously been evaluated in this same cohort, with no significant association identified [26].

Metabolic syndrome was defined according to harmonized criteria [27] as the presence of three or more of the following: central obesity (waist circumference adjusted according to ethnicity [28]), elevated triglycerides (≥1.7 mmol/L), reduced HDL cholesterol (<1.3 mmol/L), elevated blood pressure (systolic BP ≥ 130 and/or diastolic BP ≥ 85 mmHg), and/or dysglycaemia.

### 2.5. Statistical Analysis

Continuous variables were assessed for normality and are presented as median and interquartile range (IQR), while categorical variables are presented as counts and percentages. Comparisons between groups (GDM vs. controls) were performed using the median test for continuous variables, as appropriate, and the chi-square test for categorical variables.

Missingness was assessed for each variable included in the analyses. For biochemical variables with <5% missing data, missing values were imputed using the cohort mean for the corresponding variable. Given the very low proportion of missing data for individual BIA-derived variables (≤1.3%), these variables were not imputed, and analyses involving BIA measurements were conducted using available cases.

Associations between adiposity measures and cardiometabolic outcomes were evaluated using multivariable logistic regression analysis. Models were constructed for each outcome of interest, including dysglycaemia, dyslipidaemia, hypertension, metabolic syndrome and liver steatosis. A stepwise modeling approach was used to assess incremental predictive value: (i) maternal demographic and pregnancy characteristics; (ii) addition of postnatal BMI; (iii) addition of WHtR; and (iv) inclusion of body composition parameters. In the hypertension model, 13 cases in the GDM group that were previously diagnosed with chronic hypertension were excluded to restrict this analysis to women without pre-existing chronic hypertension.

Variables included in the multivariable models were selected based on biological plausibility and statistical significance in univariable analysis (*p* < 0.10). All models were adjusted for predefined confounders, including maternal age, ethnicity, family history of diabetes, history of GDM, treatment of GDM, birthweight centile, and breastfeeding status. No adjustment of statistical significance for multiple comparisons was performed.

The strength of association was expressed as odds ratios (OR) with 95% confidence intervals (CI). Model discrimination was assessed using receiver-operating-characteristic (ROC) analysis, with the area under the curve (AUC) and corresponding 95% confidence intervals reported for each model. Differences in AUCs between models were formally assessed using DeLong’s test for correlated ROC curves. A two-sided *p*-value < 0.05 was considered statistically significant. A non-significant DeLong test was interpreted as absence of evidence of a difference in discrimination rather than evidence of equivalence between models. Given the expected biological and statistical correlation between anthropometric and body-composition measures, individual body-composition parameters were evaluated separately and were not entered simultaneously into the same multivariable model. This modeling strategy further minimized the potential for unstable estimates and model overfitting.

Model calibration was evaluated using the Hosmer–Lemeshow goodness-of-fit test. A two-sided *p*-value < 0.05 was considered statistically significant. All analyses were performed using SPSS (version 29).

## 3. Results

### 3.1. Study Population

A total of 757 women were included in the study, of whom 300 (39.6%) were controls and 457 (60.4%) had been diagnosed with GDM. The postnatal follow-up assessment was conducted at a median of 5.8 (interquartile range 5.1–7.1) months after delivery in both groups, with no significant difference in timing between groups (*p* = 0.993).

### 3.2. Patient Characteristics in Early Pregnancy

At the prenatal visit at 11–13 weeks of gestation (Table 1), maternal age was similar between groups (35.6 vs. 35.5 years, *p* = 0.688). However, significant differences were observed in ethnicity distribution, with a higher proportion of Black, South Asian, and East Asian women in the GDM group (*p* < 0.001). A family history of diabetes mellitus was significantly more common in the GDM group (57.1% vs. 25.0%, *p* < 0.001), as was a history of previous GDM among parous women (30.0% vs. 2.8%, *p* < 0.001). Women with GDM, compared to controls, had higher weight (74.9 vs. 65.8 kg, *p* < 0.001), lower height (163.0 vs. 167.0 cm, *p* < 0.001), and higher BMI (27.9 vs. 23.7 kg/m^2^, *p* < 0.001).

### 3.3. Adiposity Measures at the Postnatal Visit

At the postnatal visit (Table 2), women with GDM had higher BMI, waist circumference, and waist-to-height ratio than controls (all *p* < 0.001). Body composition analysis showed greater overall and regional adiposity, including higher fat mass percentage, fat-to-fat-free mass ratio, intramuscular fat, total adipose tissue, and abdominal adipose tissue, together with lower fat-free mass (all *p* < 0.001).

At the postnatal visit, women with GDM had higher BMI, waist circumference, and waist-to-height ratio than controls (all *p* < 0.001; Table 2). Body-composition analysis showed greater overall and regional adiposity, including higher fat mass percentage, fat mass-to-fat-free mass ratio, intramuscular adipose tissue, total adipose tissue, and abdominal adipose tissue, together with lower fat-free mass (all *p* < 0.001; Table 3).

### 3.4. Comparison of Outcomes Between Groups

Women with GDM had a higher prevalence of adverse cardiometabolic outcomes at postnatal follow-up (Table 2), including dysglycaemia, hypertension, dyslipidaemia, and liver steatosis (all *p* < 0.05). No significant difference in renal dysfunction was observed between groups.

### 3.5. Prediction of Outcomes

Multivariable logistic regression analyses showed that both anthropometric and body composition measures were independently associated with all primary outcomes, including dysglycaemia (Table S1a, Figure 1A) (*p* = 0.043), dyslipidaemia (Table S1b, Figure 1B) (*p* = 0.002), hypertension (Table S1c, Figure 1C) (*p* = 0.038), metabolic syndrome (Table S1d, Figure 1D) (*p* = 0.043) and liver steatosis (Table S1e, Figure 1E) (*p* < 0.001). Models including maternal characteristics and pregnancy variables alone showed moderate discrimination (AUC range 0.648–0.811). The addition of postnatal BMI modestly improved predictive performance across outcomes, with further incremental improvement observed with WHtR, particularly for metabolic syndrome and liver steatosis.

Incorporation of body composition measures provided additional insights into risk stratification. Fat mass percentage improved discrimination compared with maternal characteristics alone *(p* = 0.023); however, no evidence of a difference in discrimination was observed compared with postnatal BMI and WHtR (*p* = 0.927). Fat mass was independently associated with hypertension (*p* = 0.029) and metabolic syndrome (*p* = 0.046) in women with prior GDM, but its inclusion did not materially enhance model discrimination beyond maternal characteristics alone, indicating limited incremental predictive value for these outcomes.

Markers of regional and ectopic adiposity showed variable predictive utility. Abdominal adipose tissue (AAT) showed superior predictive performance for liver steatosis compared with maternal characteristics and WHtR (*p* < 0.001), although its discrimination did not differ significantly from that of postnatal BMI (*p* = 0.445).

## 4. Discussion

### 4.1. Main Findings

In this prospective study of women assessed approximately 6 months postpartum, we found that those with prior GDM, compared with controls, exhibited a markedly higher burden of adverse cardiometabolic outcomes, including dysglycaemia, hypertension and liver steatosis.

Women with GDM also showed a more adverse adiposity profile, characterized by higher BMI, greater central adiposity, and increased fat mass and visceral fat depots. Body composition measures were independently associated with cardiometabolic outcomes; however, their incremental predictive value beyond conventional anthropometric indices was limited. While fat mass and abdominal adipose tissue improved discrimination compared with maternal characteristics alone, their performance was not different from BMI and waist-to-height ratio. These findings suggest that detailed body composition phenotyping enhances understanding of metabolic risk, but does not substantially improve risk prediction beyond simple clinical measures.

### 4.2. Interpretation of Results and Comparison with Previous Studies

Previous studies have shown that women with GDM exhibit a constellation of adverse cardiometabolic outcomes in the early postpartum period [29,30,31]. We confirmed this finding, showing a high burden of dysglycaemia, hypertension, and liver steatosis within the first six months after delivery. These findings are consistent with prior work showing that more than half of women with GDM develop dysglycaemia postpartum, alongside evidence of increased cardiovascular risk and hemodynamic alterations [32]. Furthermore, recent data suggest that adiposity-related organ dysfunction is highly prevalent in this population, affecting a substantial proportion of women even in the absence of overt obesity.

Adiposity has been consistently identified as a central driver of these adverse outcomes [13,33]. Previously, we and others have shown that BMI is strongly associated with postpartum cardiometabolic risk in women with prior GDM [32]. However, BMI is a crude measure that does not distinguish between fat and lean mass, nor does it capture differences in fat distribution [9,10]. This limitation is particularly relevant in populations such as women with prior GDM, in whom metabolic risk may be disproportionately influenced by visceral and ectopic fat depots rather than overall body size [9]. Indeed, emerging evidence indicates that fat distribution—especially abdominal and ectopic fat accumulation—is more closely linked to insulin resistance, hepatic steatosis, and cardiovascular dysfunction than total adiposity alone [11]. These pathophysiological mechanisms are likely to underpin the increased cardiometabolic risk observed after GDM.

In this context, our study extends previous work by incorporating detailed body composition assessment using bioelectrical impedance analysis. We showed that adiposity measures, including fat mass, abdominal adipose tissue, intramuscular fat, and fat-to-lean mass ratio, were strongly associated with adverse cardiometabolic outcomes. These findings support the concept that qualitative aspects of adiposity—particularly fat distribution and ectopic fat deposition—are key determinants of metabolic risk [34]. In particular, the association between abdominal adipose tissue and liver steatosis is biologically plausible, given the direct link between visceral fat and hepatic lipid accumulation [14].

The observed associations between adiposity and adverse cardiometabolic outcomes are likely mediated by several interrelated pathophysiological mechanisms. Central and ectopic fat depots, particularly visceral and intramuscular adipose tissue, are metabolically active and contribute to systemic insulin resistance through increased release of free fatty acids, pro-inflammatory cytokines, and adipokines [14,34]. These processes promote hepatic lipid accumulation, impaired glucose metabolism, and dyslipidaemia, which may explain the strong associations observed with liver steatosis and dysglycaemia [35]. In addition, excess adiposity contributes to hemodynamic alterations, including increased blood volume, cardiac output, and vascular resistance, which may underlie the higher prevalence of hypertension [11].

Although several body composition measures were associated with cardiometabolic outcomes, they did not show superior discrimination compared with conventional anthropometric indices in formal pairwise AUC comparisons using DeLong’s test. However, the absence of a statistically significant difference should not be interpreted as evidence of equivalence between approaches. Given the precision of the AUC estimates, small improvements in discrimination cannot be excluded and may still be clinically relevant.

### 4.3. Clinical Implications

A key contribution of the present study is the direct comparison of conventional anthropometric indices with detailed body-composition measures across multiple cardiometabolic domains within the same early postpartum population. Although previous studies have established that women with previous GDM are at increased risk of obesity, type 2 diabetes and cardiovascular disease, our findings extend this evidence by demonstrating a broader phenotype involving overall and regional adiposity, dysglycaemia, blood pressure abnormalities, metabolic syndrome and hepatic steatosis [7,32]. This multi-organ characterization is particularly relevant during the early postpartum period, which represents an important opportunity for identification of women at increased future cardiometabolic risk.

Despite demonstrating marked differences in fat mass and regional adiposity, BIA-derived measures did not demonstrate superior overall discrimination compared with conventional anthropometric indices. This distinction between phenotyping and prediction is clinically important. Detailed body-composition assessment provides insight into differences in fat mass, lean mass and regional adiposity that cannot be obtained from BMI alone; however, greater physiological detail does not necessarily translate into improved risk discrimination. In particular, WHtR incorporates waist circumference relative to body size and may therefore capture a clinically relevant component of central adiposity using a simple and readily available measurement.

These findings support a pragmatic approach to postpartum risk assessment in which BMI and WHtR can be used for initial risk stratification, including in resource-limited settings, while more detailed body-composition assessment may have a complementary role in selected women, such as those with BMI within the normal range but disproportionately high abdominal adiposity, or when detailed metabolic phenotyping is clinically or scientifically relevant. However, the present study was not designed to determine whether BIA improves risk classification in specific anthropometric subgroups, and dedicated studies are required to evaluate this possibility. No formal health-economic analysis was undertaken; therefore, conclusions regarding the relative cost-effectiveness of anthropometry and BIA cannot be drawn. Longitudinal studies are also required to determine whether detailed adiposity phenotypes provide prognostic information for longer-term cardiometabolic outcomes beyond conventional anthropometry. The association of adiposity with multiple organ-specific outcomes further supports early interventions targeting weight management and central adiposity through diet, physical activity, and weight optimization.

### 4.4. Strengths and Limitations

This study has several strengths. It includes a well-characterized cohort of women assessed prospectively in the early postpartum period, with comprehensive evaluation of anthropometric, biochemical, cardiovascular, and body composition parameters. The inclusion of both women with and without GDM allowed direct comparison of cardiometabolic risk profiles. A key strength is the detailed phenotyping of adiposity using bioelectrical impedance analysis, enabling assessment of not only overall fat mass but also fat distribution and ectopic fat depots, which are not captured by conventional measures [36]. In addition, the use of standardized definitions for cardiometabolic outcomes and a systematic modeling approach to evaluate incremental predictive value enhances the robustness and clinical relevance of the findings.

However, several limitations should be acknowledged. First, the observational design precludes inference of causality between adiposity measures and cardiometabolic outcomes. Second, assessments were performed at a single time point at a median of 5.8 months postpartum. Consequently, the study cannot determine whether the observed differences in adiposity and cardiometabolic health persist, attenuate or progress over time, nor can it establish whether early postpartum body-composition measures predict subsequent cardiometabolic disease. Longitudinal follow-up is required to characterize these trajectories and determine the long-term prognostic significance of the phenotypes identified in this study. Although timing and breastfeeding were similar between groups and breastfeeding was accounted for in the analyses, ongoing postpartum physiological changes may have influenced body composition and metabolic measurements. Thus, our findings reflect the early postpartum phenotype rather than a stable long-term cardiometabolic profile. Additionally, dysglycaemia was defined using HbA1c and glucose-based criteria in women with previous GDM, whereas equivalent glucose testing was unavailable in controls. At 5–6 months postpartum, pregnancy-related hematological changes are expected to have largely resolved. In a sensitivity analysis using glucose criteria alone, 28.4% had prediabetes and 1.8% diabetes. Despite its limitations, HbA1c provides complementary information and may identify abnormal glycemia not captured by fasting or 2 h OGTT glucose. Third, bioelectrical impedance analysis is less precise than reference methods and may be influenced by postpartum changes in hydration and fluid distribution. Although measurements followed a standardized protocol, fasting, fluid intake, and bladder voiding were not standardized, potentially introducing variability in body composition estimates. Finally, this single-center study included an ethnically and socioeconomically diverse population, with differences between women with GDM and controls. Although ethnicity was included in multivariable models, residual confounding by ethnic and socioeconomic factors cannot be excluded and should be considered when generalizing our findings.

## 5. Conclusions

In conclusion, women with prior GDM exhibit a substantial burden of cardiometabolic abnormalities in the early postpartum period, accompanied by greater overall and regional adiposity. Conventional anthropometric measures such as BMI and WHtR provided useful risk discrimination at a population level, while body-composition assessment offered additional characterization of adiposity without demonstrating superior overall discrimination in this cohort. Further studies should determine whether body-composition assessment provides incremental clinical value in selected women whose metabolic risk may not be adequately captured by conventional anthropometric measures alone. These findings support the continued use of readily available clinical measures for postpartum risk stratification, while emphasizing the importance of early intervention to reduce adiposity and mitigate long-term cardiometabolic risk.

## Figures and Tables

**Figure 1:**
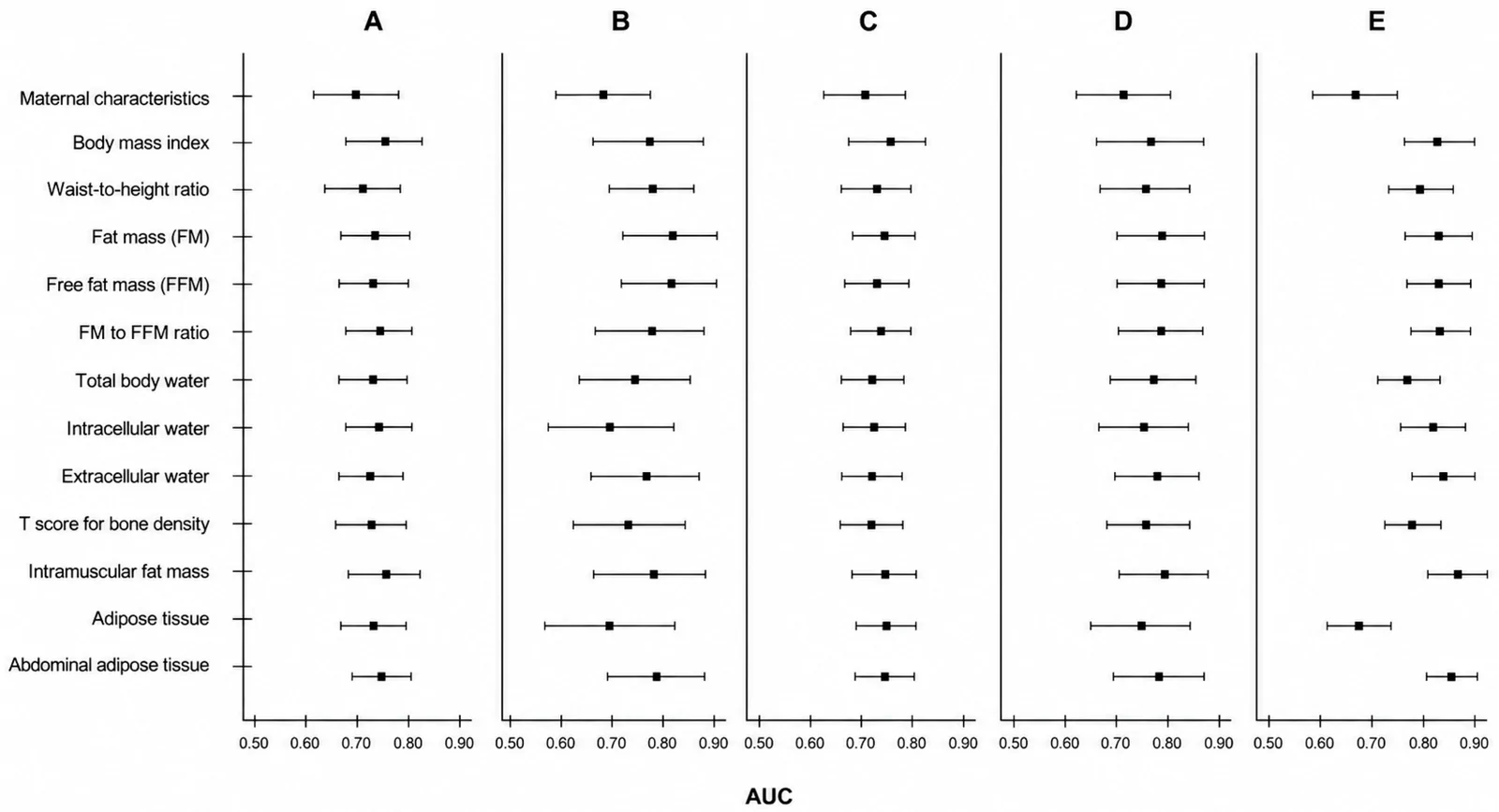
Forest plot showing area under the curve (AUC) of multivariable logistic regression models for dysglycaemia (**A**), dyslipidaemia (**B**), hypertension (**C**), metabolic syndrome (**D**) and liver steatosis (**E**). The squares represent the point estimates of AUC, and the horizontal lines indicate the corresponding 95% confidence intervals (95% CI). The dashed line represents the maternal characteristics-only AUC estimates in each model.

**Table 1:** Baseline characteristics and pregnancy outcomes of the study population in the index pregnancy.

Variables	Controls *n* = 300 (39.6%)	GDM *n* = 457 (60.4%)	*p*-Value
**Baseline characteristics at 12 weeks’ gestation in index pregnancy**			
Age (years)	35.6 (32.8–38.2)	35.5 (32.0–38.9)	0.688
Ethnicity			<0.001
White	220 (73.3)	205 (44.9)	
Black	48 (16.0)	125 (27.4)	
South Asian	14 (4.7)	77 (16.8)	
East Asian	6 (2.0)	27 (5.9)	
More than one	12 (4.0)	23 (5.0)	
Index of Multiple Deprivation quintile			0.009
Quintile 1 (<3) (most deprived)	44 (14.7)	86 (18.8)	
Quintile 2 (3–4)	76 (25.3)	134 (29.3)	
Quintile 3 (5–6)	60 (20.0)	68 (14.9)	
Quintile 4 (7–8)	64 (21.3)	63 (13.8)	
Quintile 5 (>8) (least deprived)	56 (18.7)	106 (23.2)	
Medical history			
Chronic hypertension	0 (0)	13 (2.8)	0.003
Family history			
Preeclampsia	15 (5.0)	11 (2.4)	0.055
Diabetes mellitus	75 (25.0)	261 (57.1)	<0.001
Previous pregnancies			0.071
Nulliparous	156 (52.0)	207 (45.3)	
Parous	144 (48.0)	250 (54.7)	
Parous, with previous GDM	4 (2.8)	75 (30.0)	<0.001
Parous, without previous GDM	140 (97.2)	175 (70.0)	
Parous, with previous PE	7 (4.9)	18 (7.2)	0.359
Parous, without previous PE	137 (95.1)	232 (92.8)	
Method of conception			0.412
Spontaneous	279 (93.0)	417 (91.2)	
Ovulation induction	0 (0)	2 (0.4)	
In vitro fertilization	21 (7.0)	38 (8.3)	
Weight (kg)	65.8 (59.7–75.9)	74.9 (63.9–86.5)	<0.001
Height (cm)	167.0 (162.1–172.0)	163.0 (159.0–168.0)	<0.001
BMI (kg/m^2^)	23.7 (21.3–26.9)	27.9 (23.7–32.9)	<0.001
≥30 kg/m^2^	39 (13.0)	170 (37.2)	<0.001
**Outcome of index pregnancy**			
Weight at 36 weeks (kg)	76.3 (69.7–87.0)	83.4 (71.7–95.6)	<0.001
BMI at 36 weeks (kg/m^2^)	27.8 (25.0–30.9)	31.0 (26.9–35.4)	<0.001
≥30 kg/m^2^	90 (30.0)	259 (56.7)	<0.001
Weight gain from 12 to 36 weeks (kg)	10.5 (8.0–13.0)	8.0 (4.1–11.3)	<0.001
BMI gain from 12 to 36 weeks (kg/m^2^)	3.8 (2.8–4.7)	2.9 (1.5–4.3)	<0.001
Gestational hypertension	0 (0)	33 (7.2)	<0.001
Preeclampsia	0 (0)	34 (7.4)	<0.001
Gestational diabetes control			
Diet	-	185 (40.5)	-
Metformin	-	151 (33.0)	
Insulin (+/− metformin)	-	121 (26.5)	
Gestational age at diagnosis PE/GH (weeks)	-	37.6 (35.6–39.1)	-
Gestational age at delivery (weeks)	39.7 (38.9–40.6)	38.9 (38.0–39.4)	<0.001
Birthweight percentile	45.8 (21.1–72.8)	47.3 (22.0–73.7)	0.503
≥90th	30 (10.0)	41 (9.0)	0.635

Continuous variables are expressed as median (interquartile range) and categorical variables are expressed as *n* (%). Abbreviations: GDM: gestational diabetes mellitus; PE: preeclampsia; BMI: body mass index; GH: gestational hypertension.

**Table 2:** Findings at the postnatal review of the study population.

Variables	Controls *n* = 300 (39.6%)	GDM *n* = 457 (60.4%)	*p*-Value
**Findings at postnatal study visit**			
Time to postnatal visit from birth (months)	5.8 (5.1–6.6)	5.8 (5.1–7.2)	0.993
Breastfeeding	189 (63.0)	278 (60.8)	0.548
**Anthropometric measurements**			
Weight (kg)	67.2 (59.4–78.0)	75.0 (64.5–88.4)	<0.001
BMI (kg/m^2^)	24.0 (21.6–27.7)	28.1 (24.4–32.8)	<0.001
Normal weight (BMI ≤ 24.9 kg/m^2^)	165 (55.0)	134 (29.3)	<0.001
Overweight (BMI 25.0–29.9 kg/m^2^)	88 (29.3)	144 (31.5)	
Obese type I (BMI 30–34.9 kg/m^2^)	31 (10.3)	101 (22.1)	
Obese type II and III (≥35 kg/m^2^)	16 (5.3)	78 (17.1)	
≥30 kg/m^2^	47 (15.7)	179 (39.2)	<0.001
Waist circumference (cm)	82.0 (74.0–89.6)	90.0 (81.0–100.0)	<0.001
Mid-upper arm circumference (cm)	27.0 (25.0–30.0)	29.5 (26.0–33.0)	<0.001
Waist-to-height ratio	0.49 (0.45–0.54)	0.55 (0.50–0.62)	<0.001
>0.5	125 (41.7)	330 (72.2)	<0.001
Dysglycaemia	50 (16.7)	196 (42.9)	<0.001
HbA1c (mmol/mol)	35.0 (34.0–38.0)	38.0 (35.0–41.0)	<0.001
Prediabetes	49 (16.3)	183 (40.0)	<0.001
Diabetes type 2	1 (0.3)	13 (2.8)	<0.001
Dyslipidaemia	9 (3.0)	29 (6.3)	0.039
HDL cholesterol (mmol/L)	1.7 (1.4–1.9)	1.4 (1.2–1.7)	<0.001
Serum triglycerides (mmol/L)	0.8 (0.6–1.2)	0.8 (0.6–1.2)	0.829
LDL cholesterol (mmol/L)	3.0 (2.5–3.5)	3.2 (1.6–3.7)	0.023
**Hypertension**	56 (18.7)	138 (30.2)	<0.001
Stage 1 hypertension	44 (14.7)	102 (22.3)	
Stage 2 hypertension	9 (3.0)	33 (7.2)	
**Antihypertensive medication**	7 (2.3)	15 (3.3)	
Systolic blood pressure (mmHg)	112.0 (106.5–118.4)	116.5 (108.8–124.5)	<0.001
Diastolic blood pressure (mmHg)	71.8 (67.0–76.3)	72.5 (68.0–80.0)	0.483
**Renal dysfunction**	33 (11.0)	49 (10.7)	0.904
uACR ≥ 3 (mg/mmol)	32 (10.7)	49 (10.7)	0.981
eGFR < 90 (mL/min/1.73 m^2^)	20 (6.7)	26 (5.7)	0.582
Liver assessment			
Liver stiffness measure (LSM)	4.2 (3.4–4.9)	4.3 (3.6–5.0)	0.090
Liver fibrosis with LSM ≥ 8 kPa	8 (2.7)	11 (2.4)	0.823
Liver steatosis measure (CAP)	201.0 (171.0–227.8)	224.0 (196.5–263.5)	<0.001
Liver steatosis with CAP ≥ 248 dB/m	42 (14.0)	152 (33.3)	<0.001
**Metabolic syndrome** (≥3 criteria)	12 (4.0)	32 (7.0)	0.084
Elevated waist circumference (cm)	85 (28.3)	193 (42.2)	<0.001
Serum triglycerides ≥ 1.7 (mmol/L)	32 (10.7)	49 (10.7)	0.981
HDL cholesterol < 1.3 (mmol/L)	40 (13.3)	131 (28.7)	<0.001
Elevated BP	27 (9.0)	80 (17.5)	0.001
1 criterion	99 (33.0)	197 (43.1)	<0.001
2 criteria	24 (8.0)	79 (17.3)	
3 criteria	11 (3.7)	30 (6.6)	
4 criteria	1 (0.3)	2 (0.4)	

Continuous variables are expressed as median (interquartile range) and categorical variables are expressed as *n* (%). Abbreviations: GDM: gestational diabetes mellitus; BMI: body mass index; HbA1c: glycated hemoglobin; HDL: high-density lipoprotein cholesterol; LDL: low-density lipoprotein cholesterol; uACR: urine albumin to creatinine ratio; eGFR: estimated glomerular filtration rate; LSM: liver stiffness measure; CAP: controlled attenuation parameter; BP: blood pressure.

**Table 3:** Body composition measurements of the study population.

Variables	Controls *n* = 300 (39.6%)	GDM *n* = 457 (60.4%)	*p*-Value
**Body composition measurements**			
Fat mass (FM, %)	33.0 (27.0–39.0)	39.0 (32.0–44.0)	<0.001
Fat-free mass (FFM, %)	67.0 (61.0–72.0)	61.0 (55.0–67.0)	<0.001
FM to FFM ratio (FM/FFM)	0.49 (0.39–0.64)	0.64 (0.48–0.79)	<0.001
Total body water (TBW, %)	41.0 (38.0–44.0)	39.0 (36.0–41.0)	<0.001
Intracellular water (ICW, %)	53.0 (51.0–55.0)	55.0 (52.0–58.0)	<0.001
Extracellular water (ECW, %)	47.0 (45.0–49.0)	45.0 (42.0–48.0)	<0.001
Intramuscular fat mass (IMAT, kg)	1.3 (0.9–1.8)	1.7 (1.2–2.3)	<0.001
Adipose tissue (AT, kg)	23.5 (18.9–34.4)	35.5 (25.7–49.1)	<0.001
Abdominal adipose tissue (AAT, cm^2^)	304.5 (231.2–437.9)	451.0 (319.4–632.4)	<0.001

Data are expressed as median (interquartile range). Abbreviations: GDM: gestational diabetes mellitus.

## Data Availability

The data presented in this study are available on reasonable request from the corresponding author. The data are not publicly available due to ethical and data-protection restrictions related to participant confidentiality.

## Supplementary Materials

The following supporting information can be downloaded at: https://www.mdpi.com/article/10.3390/metabo16100727/s1, Table S1: Multivariable logistic regression model for each cardiometabolic outcome; Table S1a: Multivariable logistic regression model for dysglycaemia; Table S1b: Multivariable logistic regression model for dyslipidaemia; Table S1c: Multivariable logistic regression model for hypertension (excluding cases with chronic hypertension); Table S1d: Multivariable logistic regression model for metabolic syndrome; Table S1e: Multivariable logistic regression model for liver steatosis.
