# Supplementary material for: Adiposity, Body Composition, and Cardiometabolic Outcomes in Women with and Without Gestational Diabetes
Source: Metabolites. 2026 Sep 29;16(10):727. doi: 10.3390/metabo16100727 (PMC13706881; doi:10.3390/metabo16100727)
Supplement: Supplementary file 1 [file metabolites-16-00727-s001.zip › metabolites-4494603-supplementary.pdf]

Supplementary file

Table S1: Multivariate logistic regression model for each cardiometabolic outcome.
Table S1a: Multivariate logistic regression model for dysglycaemia.

Multivariate logistic model variables included	OR (95%CI)	AUC (95%CI)
**Maternal characteristics and pregnancy outcome**		**0.699 (0.643-0.755)**
**+ Postnatal BMI (kg/m²)**	**1.068 (1.033-1.106)**	**0.729 (0.675-0.783)**
**+ Waist to height ratio**	**1.479 (0.933-2.342)**	**0.706 (0.651-0.762)**
**+ Body composition measurements**		
Fat mass (FM, %)	1.042 (1.014-1.070)	0.718 (0.663-0.773)
Free fat mass (FFM, %)	0.956 (0.931-0.983)	0.718 (0.663-0.773)
FM to FFM ratio (FM/FFM)	5.483 (2.012-14.942)	0.723 (0.668-0.777)
Total body water (TBW, %)	0.926 (0.879-0.975)	0.717 (0.662-0.772)
Intracellular water (ICW, %)	1.105 (1.042-1.172)	0.721 (0.666-0.775)
Extracellular water (ECW, %)	0.922 (0.869-0.978)	0.715 (0.660-0.770)
T score for bone density	1.233 (0.999-1.523)	0.714 (0.659-0.769)
Intramuscular fat mass (IMAT, kg)	1.652 (1.246-2.192)	0.727 (0.673-0.781)
Adipose tissue (AT, kg)	1.010 (0.999-1.022)	0.716 (0.662-0.771)
Abdominal adipose tissue (AAT, cm²)	1.002 (1.001-1.003)	0.727 (0.673-0.781)
Standard Deviation of NN intervals (SDNN, ms)	0.989 (0.982-0.997)	0.713 (0.657-0.768)
Root Mean Square of Successive Differences (RMSSD, ms)	0.993 (0.987-0.999)	0.708 (0.652-0.764)

Abbreviations: OR: odds ratio; AUC: area under the curve; CI: confidence interval; BMI: body mass index.
Dysglycaemia was defined as the presence of prediabetes or type 2 diabetes mellitus. Prediabetes was defined as one or more of the following: HbA1c 39–47 mmol/mol, fasting plasma glucose (FPG) 5.6–6.9 mmol/L, or 2-hour glucose during oral glucose tolerance test (OGTT) 7.8–11.0 mmol/L. Type 2 diabetes was defined as HbA1c ≥48 mmol/mol, fasting glucose ≥7.0 mmol/L, or 2-hour glucose ≥11.1 mmol/L.
Adjusted by age, ethnicity, family history of GDM, GDM in previous pregnancy, pharmacological treatment required for GDM control, birthweight centile and breastfeeding.

Table S1b: Multivariate logistic regression model for dyslipidaemia.

Multivariate logistic model variables included	OR (95%CI)	AUC (95%CI)
**Maternal characteristics and pregnancy outcome**		**0.648 (0.523-0.774)**
**+ Postnatal BMI (kg/m²)**	**1.108 (1.045-1.176)**	**0.731 (0.617-0.844)**
**+ Waist to height ratio**	**Undefined**	**0.738 (0.644-0.832)**
**+ Body composition measurements**		
Fat mass (FM, %)	1.118 (1.055-1.184)	0.755 (0.654-0.857)
Free fat mass (FFM, %)	0.901 (0.852-0.953)	0.750 (0.651-0.849)
FM to FFM ratio (FM/FFM)	12.992 (2.566-65.772)	0.730 (0.616-0.843)
Total body water (TBW, %)	0.866 (0.779-0.964)	0.704 (0-589-0.820)

Intracellular water (ICW, %)	1.107 (0.989-1.238)	0.665 (0.539-0.792)
Extracellular water (ECW, %)	0.828 (0.737-0.930)	0.726 (0.623-0.829)
T score for bone density	1.674 (1.117-2.510)	0.688 (0.573-0.803)
Intramuscular fat mass (IMAT, kg)	2.005 (1.267-3.172)	0.721 (0.609-0.833)
Adipose tissue (AT, kg)	1.001 (0.995-1.007)	0.663 (0.532-0.793)
Abdominal adipose tissue (AAT, cm^2)	1.003 (1.001-1.004)	0.737 (0.636-0.839)
Standard Deviation of NN intervals (SDNN, ms)	0.996 (0.981-1.011)	0.655 (0.530-0.780)
Root Mean Square of Successive Differences (RMSSD, ms)	0.999 (0.989-1.009)	0.651 (0.525-0.776)

Abbreviations: OR: odds ratio; AUC: area under the curve; CI: confidence interval; BMI: body mass index.

Dyslipidaemia was defined as the presence of high triglyceride levels (fasting serum triglycerides ≥1.7 or ≥2.3 mmol/L non-fasting), low HDL cholesterol <1.2 mmol/L and/or or use of lipid-lowering treatment.

Adjusted by age, ethnicity, family history of GDM, GDM in previous pregnancy, pharmacological treatment required for GDM control, birthweight centile and breastfeeding.

Table S1c: Multivariate logistic regression model for hypertension (excluding cases with chronic hypertension).

Multivariate logistic model variables included	OR (95%CI)	AUC (95%CI)
**Maternal characteristics and pregnancy outcome**		**0.727 (0.667-0.788)**
**+ Postnatal BMI (kg/m^2)**	**1.076 (1.038-1.115)**	**0.758 (0.701-0.815)**
**+ Waist to height ratio**	**2.915 (1.617-5.253)**	**0.747 (0.689-0.804)**
**+ Body composition measurements**		
Fat mass (FM, %)	1.064 (1.032-1.098)	0.753 (0.696-0.810)
Free fat mass (FFM, %)	0.943 (0.915-0.972)	0.751 (0.693-0.808)
FM to FFM ratio (FM/FFM)	8.217 (2.768-24.388)	0.752 (0.695-0.809)
Total body water (TBW, %)	0.904 (0.853-0.958)	0.743 (0.685-0.801)
Intracellular water (ICW, %)	1.093 (1.025-1.166)	0.743 (0.684-0.802)
Extracellular water (ECW, %)	0.911 (0.853-0.972)	0.741 (0.681-0.800)
T score for bone density	1.261 (1.004-1.585)	0.740 (0.680-0.799)
Intramuscular fat mass (IMAT, kg)	1.732 (1.288-2.330)	0.753 (0.696-0.811)
Adipose tissue (AT, kg)	1.028 (1.012-1.045)	0.762 (0.706-0.818)
Abdominal adipose tissue (AAT, cm^2)	1.002 (1.001-1.003)	0.746 (0.687-0.804)
Standard Deviation of NN intervals (SDNN, ms)	0.987 (0.978-0.997)	0.745 (0.688-0.802)
Root Mean Square of Successive Differences (RMSSD, ms)	0.989 (0.980-0.999)	0.746 (0.688-0.804)

Abbreviations: OR: odds ratio; AUC: area under the curve; CI: confidence interval; BMI: body mass index.

Hypertension was defined as systolic blood pressure ≥130 mmHg or diastolic blood pressure ≥80 mmHg, or current use of antihypertensive medication.

Adjusted by age, ethnicity, family history of GDM, GDM in previous pregnancy, pharmacological treatment required for GDM control, birthweight centile and breastfeeding.

Table S1d: Multivariate logistic regression model for metabolic syndrome.

Multivariate logistic model variables included	OR (95%CI)	AUC (95%CI)
**Maternal characteristics and pregnancy outcome**		**0.811 (0.723-0.899)**
**+ Postnatal BMI (kg/m^2)**	**1.085 (1.022-1.151)**	**0.838 (0.769-0.907)**
**+ Waist to height ratio**	**10.718 (1.401-81.983)**	**0.829 (0.751-0.908)**
**+ Body composition measurements**		
Fat mass (FM, %)	1.077 (1.019-1.139)	0.834 (0.760-0.908)
Free fat mass (FFM, %)	0.933 (0.882-0.986)	0.830 (0.757-0.904)
FM to FFM ratio (FM/FFM)	6.341 (1.316-30.558)	0.832 (0.759-0.905)
Total body water (TBW, %)	0.890 (0.799-0.992)	0.820 (0.738-0.902)
Intracellular water (ICW, %)	1.058 (0.946-1.183)	0.812 (0.729-0.894)
Extracellular water (ECW, %)	0.857 (0.761-0.964)	0.826 (0.749-0.903)
T score for bone density	1.449 (0.980-2.143)	0.820 (0.737-0.903)
Intramuscular fat mass (IMAT, kg)	1.663 (1.053-2.626)	0.828 (0.753-0.903)
Adipose tissue (AT, kg)	1.000 (0.992-1.008)	0.806 (0.716-0.896)
Abdominal adipose tissue (AAT, cm^2)	1.001 (1.000-1.003)	0.824 (0.743-0.905)
Standard Deviation of NN intervals (SDNN, ms)	0.990 (0.972-1.009)	0.824 (0.743-0.906)
Root Mean Square of Successive Differences (RMSSD, ms)	0.998 (0.986-1.010)	0.815 (0.730-0.901)

Abbreviations: OR: odds ratio; AUC: area under the curve; CI: confidence interval; BMI: body mass index.

Metabolic syndrome was defined as the presence of three or more of the following: central obesity (waist circumference adjusted according to ethnicity), elevated triglycerides (≥1.7 mmol/L), reduced HDL cholesterol (<1.3 mmol/L), elevated blood pressure (systolic BP ≥130 and/or diastolic BP ≥85 mmHg), and/or dysglycaemia.

Adjusted by age, ethnicity, family history of GDM, GDM in previous pregnancy, pharmacological treatment required for GDM control, birthweight centile and breastfeeding.

Table S1e: Multivariate logistic regression model for liver steatosis.

Multivariate logistic model variables included	OR (95%CI)	AUC (95%CI)
**Maternal characteristics and pregnancy outcome**		**0.663 (0.601-0.724)**
**+ Postnatal BMI (kg/m^2)**	**1.264 (1.202-1.329)**	**0.856 (0.814-0.898)**
**+ Waist to height ratio**	**6.556 (4.439-12.497)**	**0.770 (0.718-0.822)**
**+ Body composition measurements**		
Fat mass (FM, %)	1.201 (1.152-1.252)	0.840 (0.795-0.886)
Free fat mass (FFM, %)	0.839 (0.806-0.873)	0.839 (0.795-0.884)
FM to FFM ratio (FM/FFM)	6.339 (1.502-26.749)	0.842 (0.797-0.886)
Total body water (TBW, %)	0.763 (0.711-0.819)	0.794 (0.744-0.845)
Intracellular water (ICW, %)	1.377 (1.271-1.492)	0.827 (0.780-0.875)
Extracellular water (ECW, %)	0.692 (0.635-0.754)	0.840 (0.795-0.885)

T score for bone density	2.559 (1.984-3.301)	0.798 (0.749-0.846)
Intramuscular fat mass (IMAT, kg)	6.138 (4.103-9.183)	0.848 (0.804-0.891)
Adipose tissue (AT, kg)	1.007 (0.996-1.017)	0.706 (0.647-0.765)
Abdominal adipose tissue (AAT, cm^2)	1.008 (1.006-1.010)	0.865 (0.824-0.905)
Standard Deviation of NN intervals (SDNN, ms)	0.988 (0.979-0.997)	0.678 (0.618-0.738)
Root Mean Square of Successive Differences (RMSSD, ms)	0.992 (0.984-0.999)	0.676 (0.616-0.736)

Abbreviations: OR: odds ratio; AUC: area under the curve; CI: confidence interval; BMI: body mass index.

Liver steatosis was defined as controlled attenuation parameter or CAP ≥248 dB/m.

Adjusted by age, ethnicity, family history of GDM, GDM in previous pregnancy, pharmacological treatment required for GDM control, birthweight centile and breastfeeding
